# Effect of Solvent Removal Rate and Annealing on the Interface Properties in a Blend of a Diketopyrrolopyrrole-Based Polymer with Fullerene

**DOI:** 10.1021/acs.jpcb.2c04609

**Published:** 2022-09-19

**Authors:** Vivek Sundaram, Alexey V. Lyulin, Björn Baumeier

**Affiliations:** † Department of Mathematics and Computer Science, Eindhoven University of Technology, P.O. Box 513, 5600 MB Eindhoven, The Netherlands; ‡ Soft Matter and Biological Physics group, Department of Applied Physics, Eindhoven University of Technology, P.O. Box 513, 5600 MB Eindhoven, The Netherlands; ¶ Institute for Complex Molecular Systems, Eindhoven University of Technology, P.O. Box 513, 5600 MB Eindhoven, The Netherlands

## Abstract

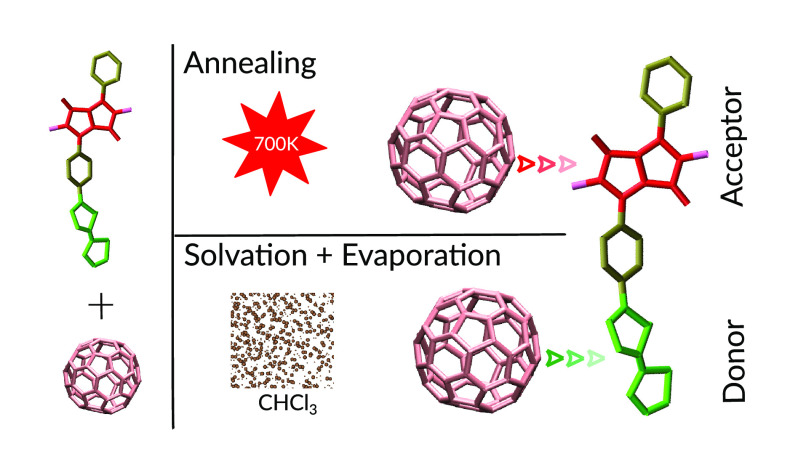

We study the effect of solvent-free annealing and explicit solvent evaporation protocols in classical molecular dynamics simulations on the interface properties of a blend of a diketopyrrolopyrrole (DPP) polymer with conjugated substituents (DPP2Py2T) and PCBM[60]. We specifically analyze the intramolecular segmental mobility of the different polymer building blocks as well as intermolecular radial and angular distribution functions between donor and acceptor. The annealing simulations reveal an increase of the glass-transition temperature of 45 K in the polymer–fullerene blend compared to that of pure DPP2Py2T. Our results show that the effective solvent evaporation rates at room temperature only have a minor influence on the segmental mobility and intermolecular orientation, characterized in all cases by a preferential arrangement of PCBM[60] close to the electron-donating substituents in DPP2Py2T. In contrast, solvent-free annealing from a liquid yields clustering of the fullerene close to the electron-withdrawing DPP, generally considered to be detrimental for application in organic solar cells. We find that the difference can be attributed to differences in the behavior of 2-hexyldecyl side-chains, which collapse toward DPP when solvent is explicitly removed, thereby blocking access of PCBM[60].

## Introduction

Polymers based on diketopyrrolopyrroles (DPP)^1−3^ are used as donor materials in polymer–fullerene organic solar cells, reaching power conversion efficiencies of around 8%.^4−7^ Their attractiveness stems from the ability to modify DPP polymers via synthetic routes that include the addition of a variety of aromatic and π-conjugated substituents to create a suitably low band gap internal donor–acceptor architecture as well as addition of specific side chains to affect solubility and structural properties following material processing.

Both the internal electronic properties of the polymer and the structural features of its blends with a fullerene acceptor upon processing on meso- and microscopic scales determine the observed power conversion efficiencies on device scale. Microscopically, the device performance is a result of how efficient several fundamental electronic processes occur, i.e., how efficiently free charges will be generated after initial absorption of photons and creation of bound (donor) electron–hole pairs and how the freed charges are transported toward the electrodes. While the latter is also determined by the existence of global pathways within the larger-scale morphology of the blend, the former is mostly influenced by an interplay of the local electronic and structural properties. Direct insights into these local properties is often difficult to obtain experimentally and is instead often sought via multiscale simulation approaches which link structural features on meso- and microscopic scales with electronic dynamics.^8−13^

The first step in such approaches is the simulation of representative structures of a polymer–fullerene blend with atomistic detail using (classical) Molecular Dynamics (MD). Obtained morphologies must as realistically as possible reflect specific structural factors important for the charge separation and transport dynamics,^14−16^ such as the segmental mobility (dihedral rotations between polymer backbone units which make or break π-conjugation) and the relative orientation of the electron-accepting fullerene with respect to the electron-donating fragments of the polymer. However, this is hardly obtainable on the same time (and length) scales involved in the experimental structure processing, in which typically a solvent is removed from a solution with donor and acceptor molecules in minutes over a hot plate. Creating a solvent–vapor interface and removing molecules that cross the interface at regular intervals^17−19^ closely emulates experimental procedures, but it can result in very long simulations, as the solvent molecules in the middle of the structure may not diffuse to the interface easily. As an alternative, we follow a procedure in which solvent is removed randomly from the solution in steps, followed by equilibration runs after each removal.^17,20^ Effective solvent removal solvent rates  have to be small enough to avoid the generation of artificial structures.

**Figure 1:**
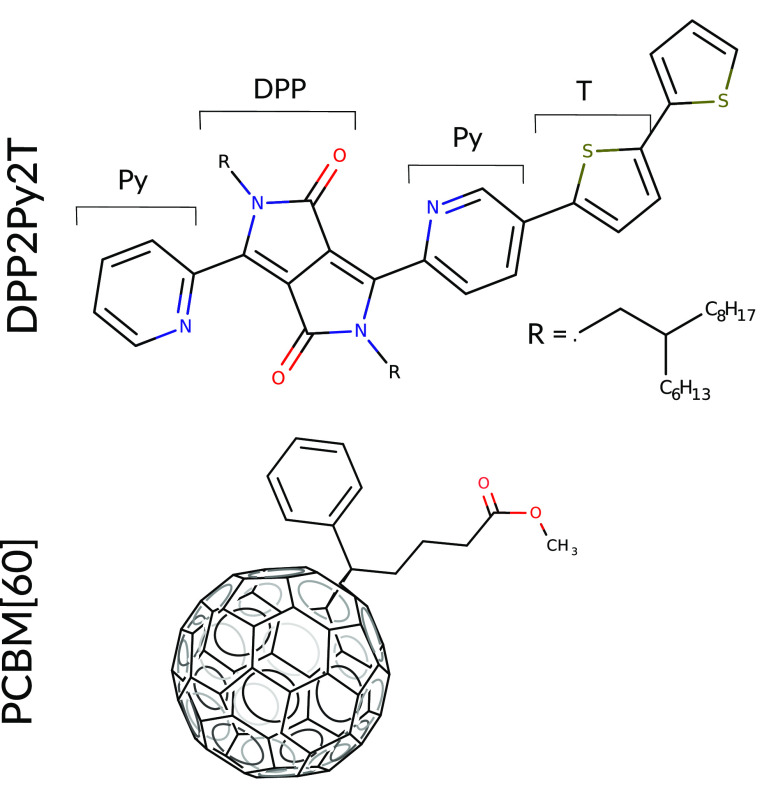
Chemical structures of DPP2Py2T (top) and PCBM[60] (bottom). For DPP2Py2T, we show the monomer building block consisting of DPP, pyridine (Py), and thiophene (T) units. R indicates the position of the 2-hexyldecyl side chain.

**Figure 2:**
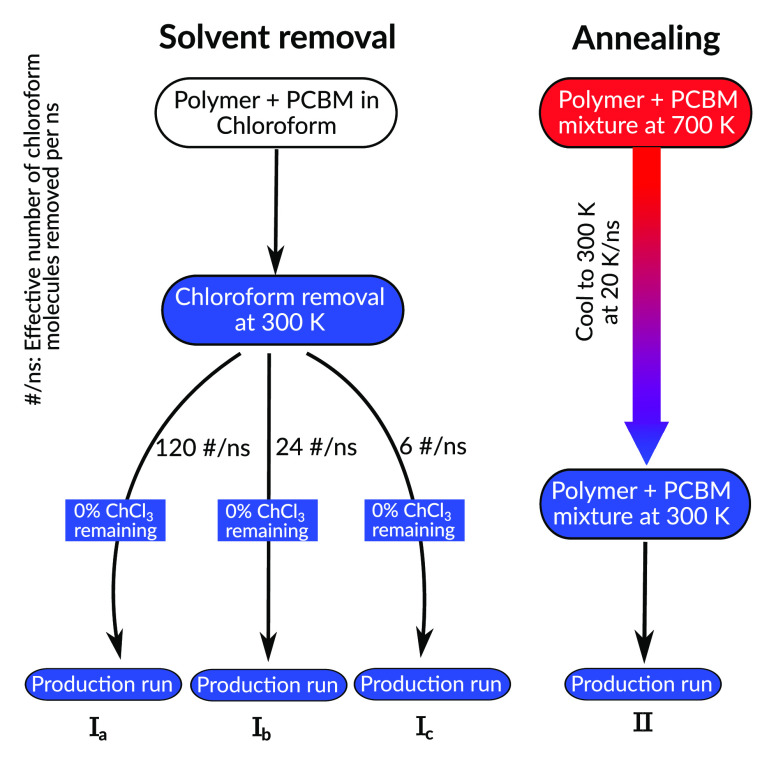
Brief summary of the two simulation protocols followed in the present study for obtaining the final structures. The first protocol envisages solvent removal at 300 K using three different removal rates 120 #/ns, 24 #/ns and 6 #/ns followed by a production run as shown in the left half of the figure. The second protocol involves first equilibrating the polymer–PCBM[60] mixture at 700 K and then cooling it to 300 K at 20 K/ns and then a production run at the end as seen in the right half of the figure. The four kinds of structures obtained at the end of the protocol are used for structural analysis.

In this work, we consider a blend of a particular DPP polymer with two aromatic pyridine (Py) and thiophene (T) substituents, respectively, in the backbone (DPP2Py2T)^21^ and PCBM[60], the chemical structures of which are shown in Figure 1. Starting from an initially solvated system in chloroform, we study the structural relaxations and final morphologies upon solvent evaporation, performed using several classical MD simulation protocols depicted in Figure 2: iterative, incremental solvent removal with different effective rates at room temperature and a solvent-free annealing from a liquid melt. The objective for this study is to scrutinize the influence of the simulation protocol on intra- and intermolecular microstructural properties. We investigate, in particular, the glass-transition temperature of the binary mixture when compared to the pure DPP2Py2T melt and the torsional mobility of the conjugated segments within DPP2Py2T with and without the presence of PCBM[60], as well as the intermolecular arrangements between DPP2Py2T and PCBM[60] via individual and combined radial and angular distribution functions as well as 3D relative density distributions. We find from the annealing simulations that the glass-transition temperature is higher for the DPP2Py2T–PCBM[60] mixture compared to pure DPP2Py2T. Segmental mobility decreases upon addition of PCBM[60] into DPP2Py2T, independent of the simulation protocol. In contrast, noticeable differences are observed for the arrangement of the fullerene acceptor with respect to the polymer obtained via solvent-free annealing or solvent evaporation, respectively. Independent of effective evaporation rate, the C_60_ units of PCBM[60] are found to be close to the π-conjugated electron-donating substituents, while annealing leads to a preferred orientation close to the electron-withdrawing DPP unit, both driven by different side-chain aggregation behavior.

This paper is organized as follows: The section Methodology contains a summary of the technical details of the general MD simulations and the specifics of the solvent evaporation and annealing protocols. Analyses of the obtained structure in terms of distribution functions are given and discussed in the Results. A brief summary concludes the paper.

## Methodology

Classical all-atom molecular dynamics simulations are performed with the Gromacs 2020.1 simulation package.^22^ The Gromos 54A7 force-field^23^ is used for PCBM[60] and chloroform molecules while a custom-made force-field, also built upon Gromos 54A7 is used to for the DPP2Py2T polymer.^24^ Gromos 54A7 parameters were obtained from the Automated force field Topology Builder (ATB, https://atb.uq.edu.au/),^25^ where bonded parameters are extracted from the Hessian of the optimized structure and partial charges through the Kollmann–Singh scheme.^26^ Starting structures were initialized with 6 polymer chains of DPP2Py2T comprising 4 monomers and 24 molecules of randomly oriented PCBM[60] in a simulation box of size 12 × 12 × 12 nm^3^. This ensured a 1:1 w/w ratio of polymer:PCBM[60] to emulate experimental concentrations.^27,28^ The idea behind choosing 4 monomer units in a single chain lies in the delocalization of the electronic density saturating at 4 repeat units when the polymer is pushed to the excited state.^13^ Two processes were subsequently followed to ensure a homogeneous blend of the polymer and PCBM[60] mixture. The first involved the solvation of the system in chloroform and then subsequent removal of chloroform at different rates. The second method involved heating the polymer PCBM[60] mixture to its melt temperature and then annealing it back to room temperature. The two methods of preparation have been shown in Figure 2. In the *NVT*/*NPT* simulations, pressure was kept constant at 1 atm using the Berendsen barostat^29^ while the temperature was maintained using the velocity-rescaling thermostat.^30^ Electrostatic interactions were calculated using particle mesh Ewald^31^ with a real space cutoff of 1.2 nm. Neighbor lists were updated every 100 time steps using a list cutoff radius of 1.0 nm. Leap frog algorithm as implemented in the md-integrator in Gromacs was used.

In the first simulation protocol, the mixture was solvated with 12000 molecules of chloroform and equilibrated first in the *NVT* ensemble at and then in the *NPT* ensemble for 200 μs at 300 K. Due to the high solvent concentration, the density saturated to chloroform density of 1.517 g/cm^3^. The next step is solvent evaporation, which we model by removing chloroform from the solution in batches containing *N*_r_ molecules with a time interval *T*_r_ between removal steps. During the whole process, the system is simulated in the *NPT* ensemble described above. As the concentration of DPP2Py2T and PCBM[60] in the solution is very low initially, the stepwise procedure is performed in two stages: In the first stage, nine removal steps are executed with ; i.e., 10% of the initially present solvent molecules are removed at each step, until at the end only 1200 chloroform molecules are left. This is followed by stage two, comprising 10 steps with . As also listed in Table 1, we perform simulations at 300 K with different lengths of the time intervals *T*_r_ during the two stages. They are chosen to yield three different total simulation times of the evaporation procedure of 100 ns, 500 ns, and 2 μs, corresponding to constant effective evaporation rates  of 120 #/ns (I_a_), 24 #/ns (I_b_) and 6 #/ns (I_c_), respectively. Three different  have been chosen to investigate the influence of the simulated speed of evaporation on the structural features in the final room-temperature morphologies.

**Table 1:** Parameters for the Three Different Solvent Evaporation Simulations: Number of Removed Chloroform Molecules per step *N*_r_ in Stages 1 and 2, the Respective Lengths of the Time Intervals *T*_r_, Total Time of the Simulated Evaporation , and the Effective Evaporation Rate  in molecules/ns

	*N*_r_(1)	*T*_r_(1)	*N*_r_(2)	*T*_r_(2)	T_r_^total^	*k*_r_^eff^
I_a_	1200	10 ns	120	1 ns	100 ns	120 ns^–1^
I_b_	1200	50 ns	120	5 ns	500 ns	24 ns^–1^
I_c_	1200	200 ns	120	20 ns	2 μs	6 ns^–1^

As an alternative to the explicit solvent evaporation simulations (I_a_–I_c_), we also perform a solvent-free simulation of the DPP2Py2T–PCBM[60] blend. In this annealing approach, the initial mixture is first equilibrated at 700 K (which, as will be confirmed later, is about 250 K above the glass transition temperature) and then cooled to 300 K with a cooling rate of 20 K/ns (structure II).

For the structural analysis, *NPT* production runs of 20 ns are performed for the systems obtained via protocols I_a,b,c_ and II. The density of the final production runs stabilized at 1285 kg/m^3^ for Type I_a,b,c_ structures and 1305 kg/m^3^ for Type II structures. Note that due to the random selection in the removal of the chloroform molecules, results from five independent simulations runs are averaged in the analysis of systems I_a_–I_c_. Over the last 10 ns of the respective runs, the intermolecular radial distribution function (RDF), angular distribution (ADF) function, and the combined distribution function (CDF) between each unit of DPP2Py2T (DPP, Py, T) and C_60_ part of PCBM[60] are determined. Also, the preferred side-chain orientation with respect to the DPP block of DPP2Py2T is visualized. All analysis and postprocessing was carried out using the Trajectory Analyzer and Visualizer (TRAVIS) code.^32,33^

## Results

### Glass Transition

**Figure 3:**
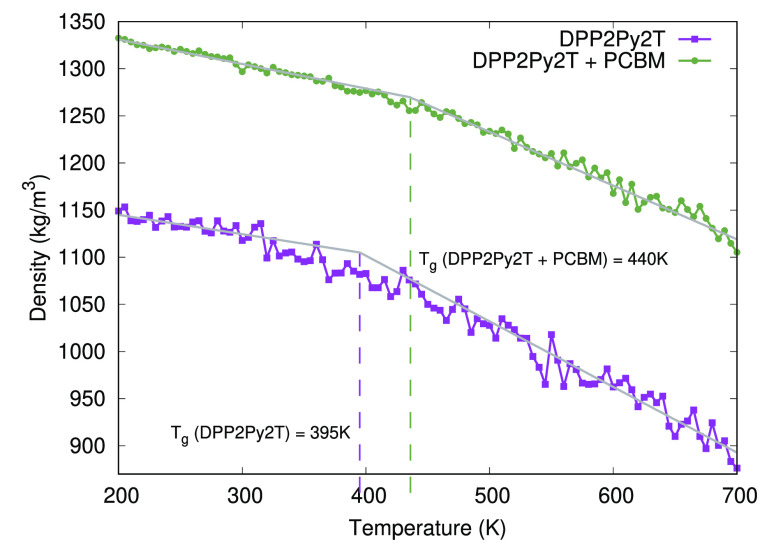
Density variation of pure DPP2Py2T melt and the DPP2Py2T–PCBM[60] mixture with temperature. Straight lines show linear fitting of the simulated data at high and low temperatures, with the glass-transition temperature of DPP2Py2T melt as *T*_g_ = 395 ± 20 K and for mixture as *T*_g_ = 440 ± 20 K obtained from their intersection.

Before we turn to the analysis of the inter- and intramolecular structural features of the four DPP2Py2T–PCBM[60] blends from I_a,b,c_ and II, we briefly discuss the dependence of the blend density on temperature during the annealing process (II), as shown in Figure 3. Initially, at 700 K, the DPP2Py2T melt has a density of 870 kg/m^3^, which increases to 1150 kg/m^3^ at 200 K while the blend density goes from 1110 kg/m^3^ at 700 K to 1330 kg/m^3^ at 200 K, which is considerably higher than the melt density due to the high PCBM[60] density. One can clearly see the glass transition, whose temperature *T*_g_ is estimated from linear fits in the temperature intervals 200–300 K and 550–700 K as 440 ± 20 K. This is significantly higher than the glass-transition temperature of 395 ± 20 K for a pure DPP2Py2T polymer melt due to nonbonded interactions between DPP2Py2T and PCBM[60]. Such an increase of the glass-transition temperature by 40–50 K as a result of the addition of PCBM[60] is consistent with previous studies of blends with similar weight fraction of fullerene and a P3HT polymer.^34,35^

### Segmental Mobility

**Figure 4:**
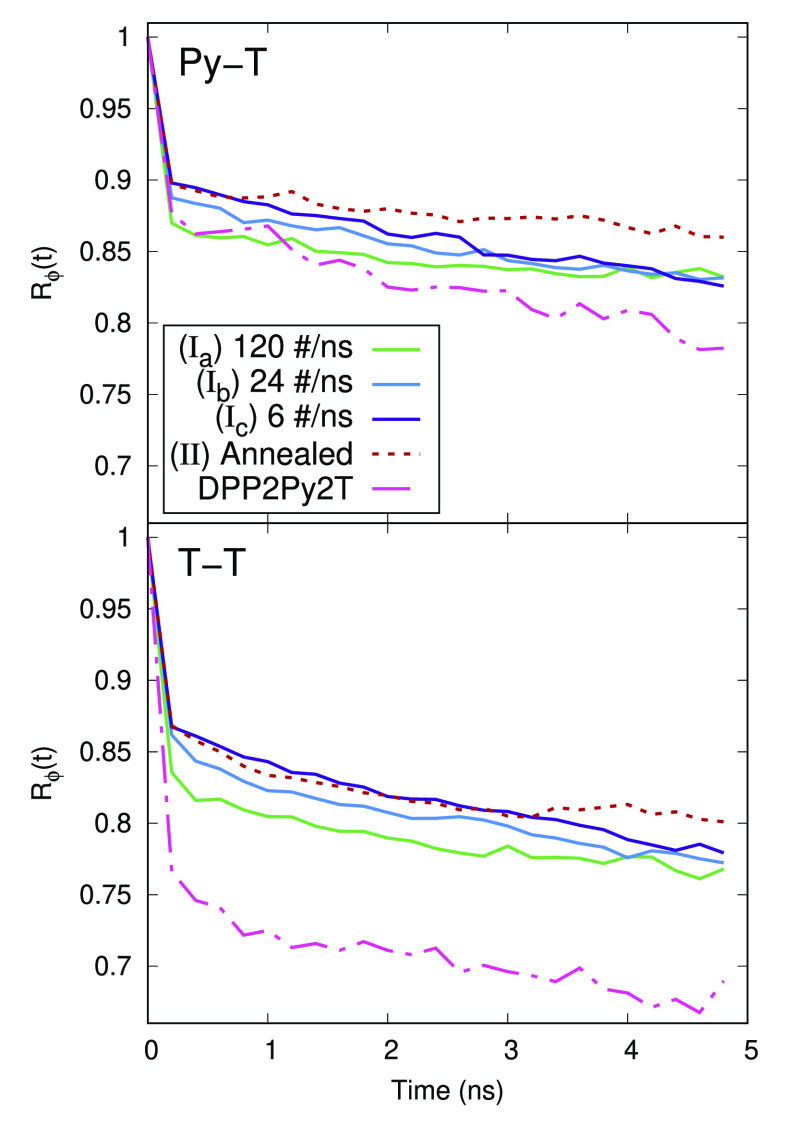
Torsional autocorrelation function for the Py–T (top panel) and T–T (bottom panel) units in the DPP2Py2T–PCBM[60] blend, obtained by solvent evaporation (solid lines) and solvent-free annealing (dashed line). A reference TACF for the pure DPP2Py2T system is shown by a dash-dotted line.

Torsional autocorrelation functions (TACFs) for the torsional angle between pyridine-thiophene (Py–T) and thiophene-thiophene (T–T) units in the backbone of DPP2Py2T provide insight into the intramolecular dynamics in the four final morphologies of the DPP2Py2T–PCBM[60] mixture. Specifically, we study the conformational mobility using the autocorrelation function for dihedrals.^36^
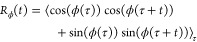
1Here ϕ(*t*) is the angle between the normal vectors of the Py and T molecular planes at time *t*. The TACF is computed for a time span of 5 ns, averaged over 40 different starting times (τ). To understand the impact of the presence of PCBM[60] on the mobility of the different segments of the polymer, a reference simulation was performed with polymer only at 300 K. Figure 4 shows the TACF for the Py–T and T–T units for both simulation protocols and DPP2Py2T polymer only. First, one notices that the relaxations can be classified into two distinct parts- a rapid relaxation that occurs within the first 200 ps characterized by torsional vibrations around the minimum and then later a second decay with a longer time scale corresponding to *cis*–*trans* flipping between the respective units. The time-scales for the slower relaxation times are obtained from exponential fits to the TACF data and shown in Table 2.

**Table 2:** Relaxation Times for the Py–T and T–T Connections as Obtained from the Variable Solvent Removal Rates and Annealing Procedures along with Only DPP2Py2T Structures at 300 K

	Py–T (ns)	T–T (ns)
I_a_ (120 ns^–1^)	100	50
I_b_ (24 ns^–1^)	60	40
I_c_ (6 ns^–1^)	60	40
II (annealing)	110	60
DPP2Py2T	40	30

The structures without the presence of PCBM[60] (dash-dotted lines) relax to a greater extent within the first 200 ps and this phenomena is more prominent for T–T link. The relaxation times for both Py–T and T–T units are much smaller in the absence of PCBM[60] as seen from Table 2. We notice that the presence of PCBM[60] increases the relaxation times from 50 to 60–110 ns for Py–T units and from 30 to 40–60 ns for the T–T units. In a nutshell, it can be concluded that the nonbonded interactions between the PCBM[60] and DPP2Py2T in the system act as a binder to restrict the intramolecular segmental mobility. This, in turn, can be beneficial for charge transport as it helps maintain conjugation within the polymer backbone. Also, for structure II that was obtained from annealing (red dotted lines), the TACF attains a saturation point faster than structures I_a-c_ thereby revealing a higher relaxation time.

Py–T correlation decays are seen in Figure 4 at slower rate as compared to T–T correlation, which can be attributed to the lower torsional barrier for T–T connection compared to the Py–T connection.

### Fullerene Distribution around the Polymer

**Figure 5:**
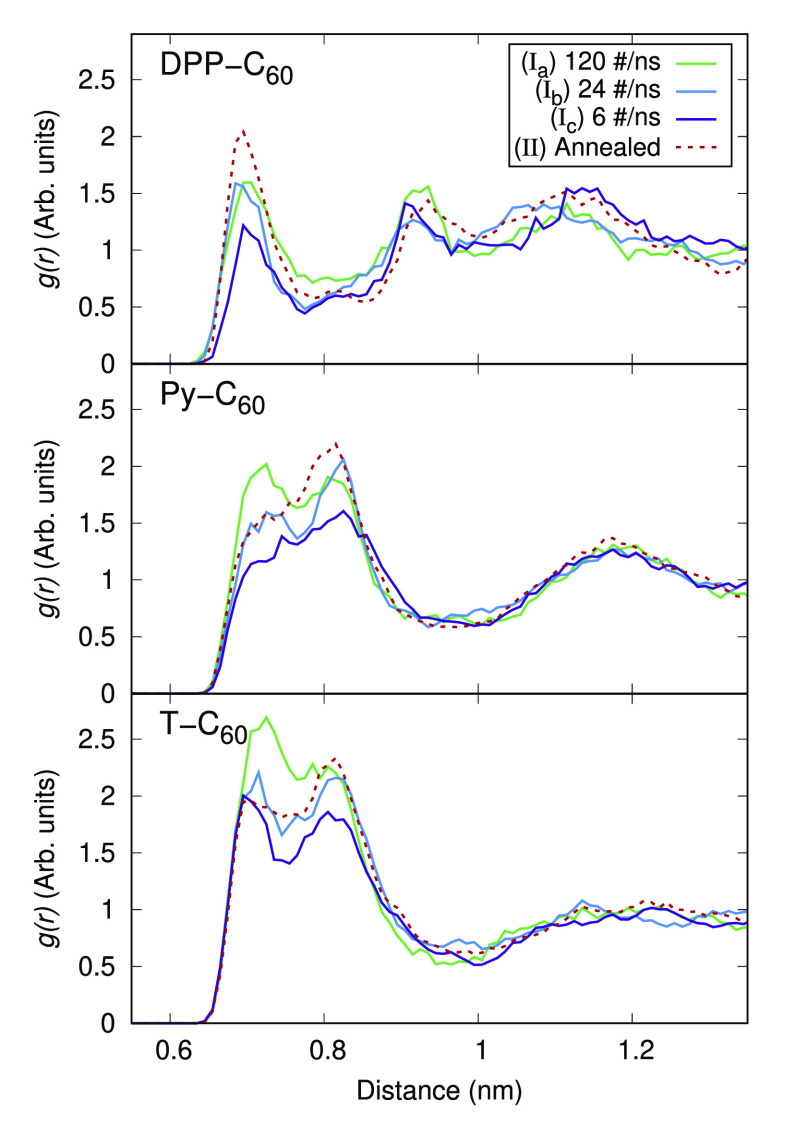
Radial distribution function *g*(*r*) between the centers-of-geometry of C_60_ and DPP (top panel), pyridine (middle panel), and thiophene (bottom panel) obtained using three different effective solvent evaporation rates (solid lines) and solvent-free annealing (dashed line), respectively.

Having seen the intramolecular structural correlation between pyridine and a thiophene unit and two thiophene units in the polymer system we intend to explore the intermolecular orientation between the individual units in the polymer and the C_60_ unit of PCBM[60]. As stated earlier, the relative orientation of the PCBM[60] with the individual units of the polymer is, in principle, important for charge transfer phenomena as discussed in the Introduction earlier. To characterize the orientation, we use three distribution functions, radial *g*(*r*), angular *g*(θ), and a combined distribution function *g*(*r*,θ). In these calculations, we do not consider the hydrogen atoms in the system and also ignore the side chain on the polymer and PCBM[60]. Also, for point to point calculations, we will only consider the center of geometry for all units, so the distances in *g*(*r*) are the distance between the center of geometry of the respective units. The results for the radial distribution function can be seen in Figure 5. The first coordination for the C_60_ unit w.r.t each unit of the polymer is roughly at about the 0.8 nm. The system obtained after annealing showed more aggregation of C_60_ around the DPP unit of the polymer as visible from a higher peak height. For other simulation pathways, I_a_–I_c_, the DPP unit shows dispersed peaks indicating no preferred distance between the DPP–C_60_ pairs. For the Py–C_60_ pair, two peaks are visible at the first coordination distance of 0.8 nm and at the second coordination distance of 1.2 nm. The first coordination distance between the thiophene and C_60_ units are split into two peaks. The system obtained from annealing shows a larger peak at 0.8 nm while the systems obtained from solvent removal show a more prominent peak at 0.75 nm. A general observation is that the C_60_ molecule showed a preferential alignment to the thiophene, pyridine, and DPP units in decreasing order as seen from the number of observable peaks and their respective heights. However, in the case when the system was cooled from 700 K, the relative height of the all peaks (red curves) are similar, indicating that PCBM[60] in these structures does not have a clear preference unlike the structures attained by solvent removal where PCBM[60] shows aggregation around the electron-donating units of the polymer. Hence, we can conclude that different removal rates for solvent molecules do not explicitly affect the distance distribution of fullerenes around the polymer backbone. However, annealing from a higher temperature has a more prominent effect of C_60_ aggregation around the DPP fragment of the polymer.

**Figure 6:**
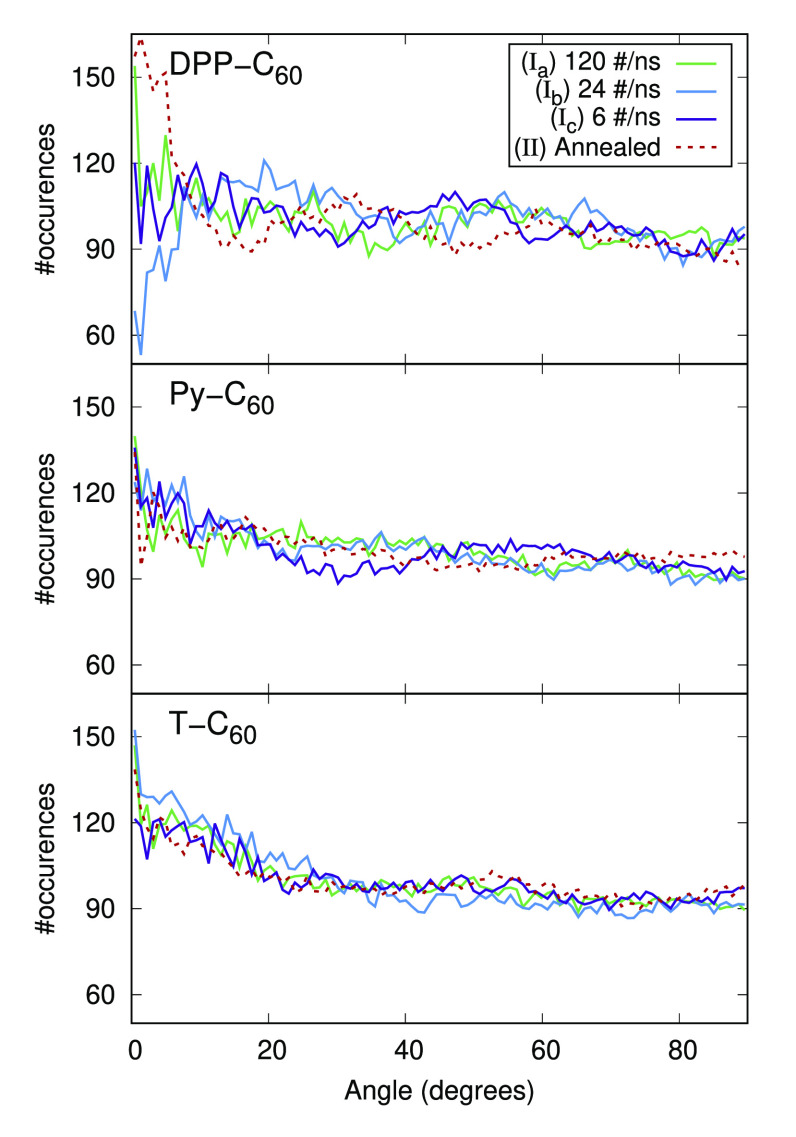
Angular distribution function defined between normals of the molecular plane for DPP (top panel), pyridine (middle panel), and thiophene (bottom panel), and a vector connecting centers-of-geometry of the polymer unit and C_60_ obtained from using three different effective solvent evaporation rates (solid lines) and solvent-free annealing (dashed line), respectively.

For the angular and cumulative distribution functions a vector was defined normal to the plane of the polymer unit under study similar to the one we defined in the Segmental Mobility subsection. A second vector is defined by connecting the center of geometries of the polymer unit and C_60_, which is the same as we have used to compute the radial distribution function *g*(*r*). We calculate the angle between this normal vector and the connecting vector. For instance, 0° represents a fullerene molecule exactly above the plane of the unit of the polymer and 90° represents the fullerene molecule lying within the fragment plane. Cone correction is also applied to maintain consistency of the distribution. The angular distribution in Figure 6 shows an almost flat line with a little monotonic decrease for Py–C_60_ and T–C_60_ pairs as the angle increases from 0° to 90°. Most fluctuations are noticed for the DPP–C_60_ pair. It is worthy to note that having a C_60_ unit on either side of the polymer plane is considered similar, which is why the study was done only up to 90°.

**Figure 7:**
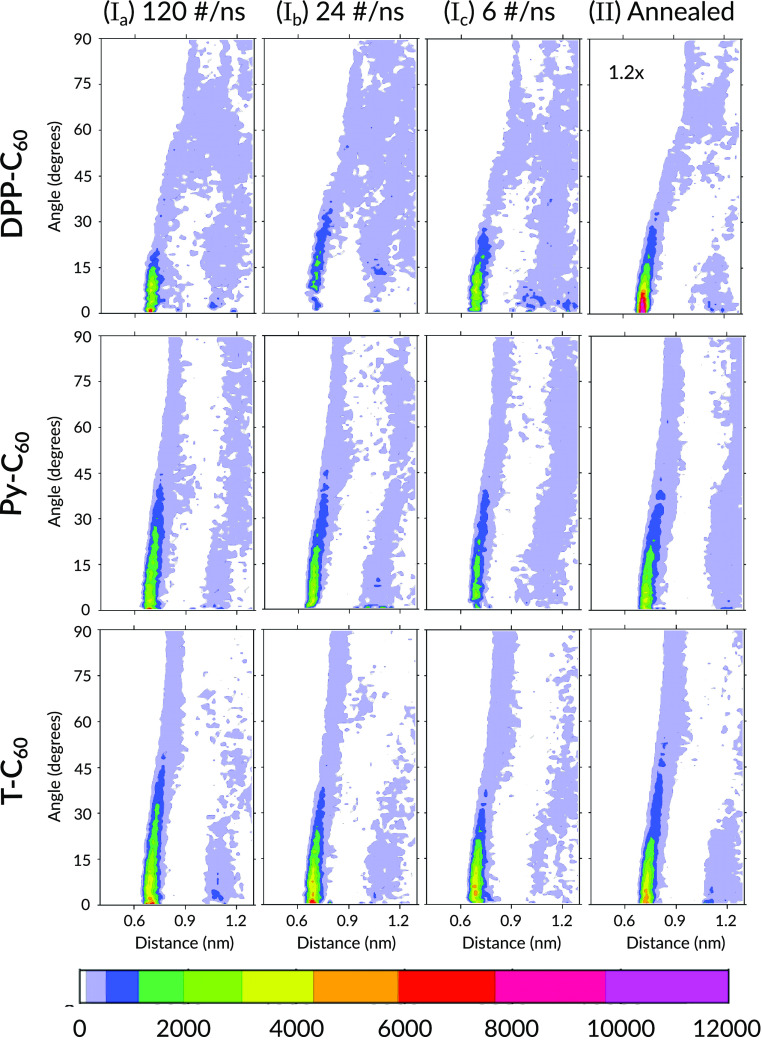
Combined distribution function involving the radial distribution on the horizontal axes and angular distribution on the vertical axes show the corresponding angle distributions. The color coding represents the chances of finding a particular combination of the two distribution functions. Note that for the DPP–C_60_ pair obtained from annealing, the color scale has been scaled down by a factor of 1.2 to match with the color scheme of the other cases.

In Figure 7, we show the results for a combined distribution function of different units within the polymer and C_60_ molecule. The radial and angular distributions present an idea about the relative distance and orientation of the polymer units and the C_60_ molecule separately; however, a combined study of the two tells us which intermolecular positions are the most favorable. As a guide to Figure 7, we need to note that each column of the figure contains plots for different simulation protocols while each row contains the plots for arrangement of different units of the DPP2Py2T polymer w.r.t. the C_60_ unit of PCBM[60]. It is also to be noted that the color scale of the plot for the DPP–C_60_ pair obtained from the annealing procedure has been scaled down by a factor of 1.2 to match the color scheme of the plots for the four other simulation protocols. The polymer units were taken as the reference while the C_60_ units around it were observed.

Observing the first row of plots in Figure 7, we immediately see a distinct bright red spot between 0.6 and 0.9 nm at 0° for the system cooled from 700 K. This clearly indicates an accumulation of C_60_ units around the DPP units. In the same row the system cooled from 700 K also shows patches of orange at the same spot which led us to conclude that higher temperatures lead to C_60_ accumulation around the DPP unit which is detrimental to the type of arrangement preferred for solar cell functioning. Now looking at Py–C_60_ and T–C_60_ combinations, we notice a large band of white space between 0.8 and 1 nm. This shows the absence of C_60_ units at those distances for any angular orientation. This is consistent with the dip in the peaks for their respective *g*(*r*) as seen in Figure 5. This low density region (white spaces) is credited to the excluded volume for PCBM[60]. That is why it can be seen that the higher the concentration of C_60_, the more the excluded volume is, thereby indicating more white spaces as seen in Figure 7.

### Influence of Side-Chain Alignment

In this section we investigate the relative density of the C_60_ unit of PCBM[60] and the side-chain (2-hexyldecyl) around the DPP unit of the polymer. The center of geometry of the DPP unit is positioned at the origin and the center of geometries of C_60_ and individual branches of the side-chain are observed in this scenario.

**Figure 8:**
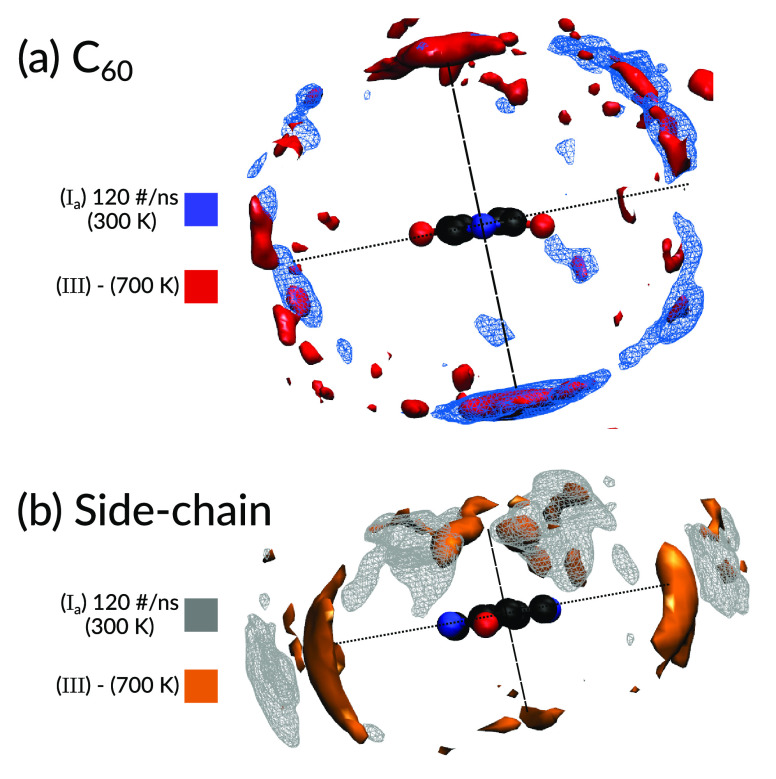
Relative densities of (a) C_60_ and (b) side-chain around the DPP unit of the polymer is shown when visualized from the polymer plane. The filled bubbles (red and orange indicate density around structure II that was cooled from 700 K while the wired mesh (blue and gray) indicate density around structure I_a_ that was maintained at 300 K and had a chloroform removal rate of 120 #/ns.

As we see in Figure 8a, the C_60_ molecules show a larger density exactly above and below the plane of the DPP unit. However, one can notice that for structure (I_a_) (blue mesh) the density of C_60_ is limited to only one side of the DPP plane while for structure (II) (solid red) there is presence of C_60_ density on both sides of the DPP plane. As we do not distinguish between 0° and 180° while calculating angle distribution, it can intuitively understood why this leads to higher concentration of C_60_ around DPP for structure II, as observed in Figure 7 as well. This brings about the question as to how the side-chains present on the DPP would be oriented to facilitate such an alignment.

The relative density of the side-chain around the DPP unit of the polymer can be seen in Figure 8b as viewed from the molecular plane. It is noteworthy to remember that the side-chains are attached to the nitrogen atom of the DPP unit as seen in Figure 1. Also the side-chain contains two branches containing 6 and 8 carbon atoms in each branch, respectively, as shown in Figure 1. Hence, the two branches are treated separately by considering the center of geometry of each branch individually. In the end, the relative density of both branches are added to reveal the total side chain density around the DPP unit. We can see in Figure 8b that in case of structure II (orange solid) the side-chains pack closer to the DPP unit within the plane and very very little little density is observed above and below the DPP plane indicating a constricted packing which facilitates the positioning of C_60_ molecule above and below the DPP plane. However, for structure I_a_ (gray mesh), the side chains are farther away from the DPP unit indicating a more open structure and also the two branches of the side-chain pack distinctly away from each other in perpendicular planes. This inherently blocks a substantial region above the DPP unit, which makes it inaccessible to the C_60_ unit.

The solvent concentration and orientation around the DPP unit influences the packing of side-chains. The purpose of side-chains in these polymers is enhancing solubility to facilitate ease of processing. In simulations, the longer the polymer is exposed to a good solvent (chloroform in this case) the greater is the extent of side-chain expansion which in-turn reduces the available space around the DPP unit (see Figure S1 of the Supporting Information).

Finally, we note that a local arrangement of PCBM[60] near the electron-donating substituents of the polymer is also commonly considered as the most favorable arrangement, inferred from the fact that DPP2Py2T/PCBM[60] mixtures have been used in active layers of polymer-based solar cells.^1^ Here, (efficient) charge generation requires the easy formation of charge transfer states after photon absorption, with the hole on the internally electron-donating substituents of the polymer and the electron on fullerene. The formation of such states can be considered energetically unlikely between the internally electron-accepting DPP and PCBM[60]. While this observation provides merely a very indirect qualitative link between the simulations and real systems, it suggests that the simulation protocol (II) based on annealing of a melt above the glass transition yields qualitatively wrong local arrangements and that explicit solvent effects need to be accounted for in a reliable simulation protocol. Such information is relevant for multiscale modeling approaches that aim at gaining microscopic insight into the charge separation/generation processes in the DPP (and possibly other push–pull architecture) polymer heterojunctions with PCBM,^8−13^ which rely on an accurate atomistic model of its morphology.

## Conclusions

In this paper, we analyze the relative orientation of the C_60_ unit of PCBM[60] around different units of DPP2Py2T polymer for two simulation protocols involving a variation in solvent removal rate and annealing from melt. The glass-transition temperature for the polymer-PCBM[60] mixture was found to be 440 ± 20 K, which was expectedly higher than *T*_g_ for DPP2Py2T only. The intramolecular segmental mobility was lowered upon addition of PCBM[60] as seen from the higher relaxation times owing to the nonbonded interaction between the PCBM[60] and DPP2Py2T. As for the local orientation of C_60_ around the polymer, the solvent removal rate did not influence as much as the temperature variation did. We see that the C_60_ unit preferred a closer vicinity to the DPP unit of the polymer when cooled from 700 K. This was assisted by the closer packing of the side-chains in the plane of the DPP unit, thereby leaving available space for C_60_ to come close to DPP.

## Supporting Information Available

(i) Table with all simulation parameters, (ii) figure of the combined angular and radial distribution function of chloroform and DPP for different solvent concentrations during the evaporation process, and (iii) comparison of the local arrangements for fast solvent evaporation for a selected system with double the number of polymer and fullerene molecules (PDF)

## References

[ref1] Wienk M. M.; Turbiez M.; Gilot J.; Janssen R. A. Narrow-bandgap diketo-pyrrolo-pyrrole polymer solar cells: The effect of processing on the performance. Adv. Mater. 2008, 20, 2556–2560. 10.1002/adma.200800456.

[ref2] Chandran D.; Lee K.-S. Diketopyrrolopyrrole: A Versatile Building Block for Organic Photovoltaic Materials. Macromol. Res. 2013, 21, 272–283. 10.1007/s13233-013-1141-3.

[ref3] Li W.; Hendriks K. H.; Wienk M. M.; Janssen R. A. Diketopyrrolopyrrole Polymers for Organic Solar Cells. Acc. Chem. Res. 2016, 49, 78–85. 10.1021/acs.accounts.5b00334. 26693798

[ref4] Choi H.; Ko S. J.; Kim T.; Morin P. O.; Walker B.; Lee B. H.; Leclerc M.; Kim J. Y.; Heeger A. J. Small-bandgap polymer solar cells with unprecedented short-circuit current density and high fill factor. Adv. Mater. 2015, 27, 3318–3324. 10.1002/adma.201501132. 25899940

[ref5] Ashraf R. S.; Meager I.; Nikolka M.; Kirkus M.; Planells M.; Schroeder B. C.; Holliday S.; Hurhangee M.; Nielsen C. B.; Sirringhaus H.; et al. Chalcogenophene comonomer comparison in small band gap diketopyrrolopyrrole-based conjugated polymers for high-performing field-effect transistors and organic solar cells. J. Am. Chem. Soc. 2015, 137, 1314–1321. 10.1021/ja511984q. 25547347

[ref6] Hendriks K. H.; Heintges G. H.; Gevaerts V. S.; Wienk M. M.; Janssen R. A. High-molecular-weight regular alternating diketopyrrolopyrrole-based terpolymers for efficient organic solar cells. Angew. Chem., Int. Ed. 2013, 52, 8341–8344. 10.1002/anie.201302319. 23794318

[ref7] Heinrichová P.; Pospíšil J.; Stříteský S.; Vala M.; Weiter M.; Toman P.; Rais D.; Pfleger J.; Vondráček M.; Šimek D.; et al. Diketopyrrolopyrrole-Based Organic Solar Cells Functionality: The Role of Orbital Energy and Crystallinity. J. Phys. Chem. C 2019, 123, 11447–11463. 10.1021/acs.jpcc.9b01328.

[ref8] Wehner J.; Baumeier B. Multiscale simulations of singlet and triplet exciton dynamics in energetically disordered molecular systems based on many-body Green’s functions theory. New J. Phys. 2020, 22, 033033 10.1088/1367-2630/ab7a04.

[ref9] Wehner J.; Brombacher L.; Brown J.; Junghans C.; Tirimbo G.; Çaylak O.; Khalak Y.; Madhikar P.; Baumeier B. Electronic Excitations in Complex Molecular Environments: Many-Body Green’s Functions Theory in VOTCA-XTP. J. Chem. Theory Comput. 2018, 14, 6253–6268. 10.1021/acs.jctc.8b00617. 30404449 PMC6293448

[ref10] Li J.; D’Avino G.; Duchemin I.; Beljonne D.; Blase X. Combining the Many-Body GW Formalism with Classical Polarizable Models: Insights on the Electronic Structure of Molecular Solids. J. Phys. Chem. Lett. 2016, 7, 2814–2820. 10.1021/acs.jpclett.6b01302. 27388926

[ref11] De Vries X.; Friederich P.; Wenzel W.; Coehoorn R.; Bobbert P. A. Full quantum treatment of charge dynamics in amorphous molecular semiconductors. Phys. Rev. B 2018, 97, 075203 10.1103/PhysRevB.97.075203.

[ref12] Rühle V.; Lukyanov A.; May F.; Schrader M.; Vehoff T.; Kirkpatrick J.; Baumeier B.; Andrienko D. Microscopic simulations of charge transport in disordered organic semiconductors. J. Chem. Theory Comput. 2011, 7, 3335–3345. 10.1021/ct200388s. 22076120 PMC3210523

[ref13] Tirimbò G.; Sundaram V.; Çaylak O.; Scharpach W.; Sijen J.; Junghans C.; Brown J.; Ruiz F. Z.; Renaud N.; Wehner J.; et al. Excited-state electronic structure of molecules using many-body Green’s functions: Quasiparticles and electron-hole excitations with VOTCA-XTP. J. Chem. Phys. 2020, 152, 114103 10.1063/1.5144277. 32199411

[ref14] Guerrero A.; Garcia-Belmonte G. Recent advances to understand morphology stability of organic photovoltaics. Nano-Micro Lett. 2017, 9, 1–16. 10.1007/s40820-016-0107-3. PMC6223777 30460307

[ref15] Savikhin V.; Babics M.; Neophytou M.; Liu S.; Oosterhout S. D.; Yan H.; Gu X.; Beaujuge P. M.; Toney M. F. Impact of Polymer Side Chain Modification on OPV Morphology and Performance. Chem. Mater. 2018, 30, 7872–7884. 10.1021/acs.chemmater.8b03455.

[ref16] Grozema F. C.; Van Duijnen P. T.; Berlin Y. A.; Ratner M. A.; Siebbeles L. D. Intramolecular charge transport along isolated chains of conjugated polymers: Effect of torsional disorder and polymerization defects. J. Phys. Chem. B 2002, 106, 7791–7795. 10.1021/jp021114v.

[ref17] Larsen A. S.; Ruggiero M. T.; Johansson K. E.; Zeitler J. A.; Rantanen J. Tracking dehydration mechanisms in crystalline hydrates with molecular dynamics simulations. Cryst. Growth Des. 2017, 17, 5017–5022. 10.1021/acs.cgd.7b00889.

[ref18] Negi V.; Lyulin A.; Bobbert P. Solvent-Dependent Structure Formation in Drying P3HT:PCBM Films Studied by Molecular Dynamics Simulations. Macromol. Theory Sim. 2016, 25, 550–558. 10.1002/mats.201600075.

[ref19] Tsige M.; Grest G. S. Molecular dynamics study of the evaporation process in polymer films. Macromolecules 2004, 37, 4333–4335. 10.1021/ma049509v.

[ref20] Tabe H.; Kobayashi K.; Fujii H.; Watanabe M. Molecular dynamics simulation of evaporation coefficient of vapor molecules during steady net evaporation in binary mixture system. Int. J. Heat Mass Transfer 2022, 188, 122663 10.1016/j.ijheatmasstransfer.2022.122663.

[ref21] Hendriks K. H.; Wijpkema A. S.; Van Franeker J. J.; Wienk M. M.; Janssen R. A. Dichotomous Role of Exciting the Donor or the Acceptor on Charge Generation in Organic Solar Cells. J. Am. Chem. Soc. 2016, 138, 10026–10031. 10.1021/jacs.6b05868. 27452683

[ref22] Bekker H.; Berendsen H.; Dijkstara E.; Achterop S.; Vondrumen R.; Vanderspoel D.; Sijbers A.; Keegstra H.; Renardus M. GROMACS - A parallel computer for molecular-dynamics simulations; World Scientific Publishing: Singapore, 1993; pp 252–256.

[ref23] Reif M. M.; Winger M.; Oostenbrink C. Testing of the GROMOS force-field parameter set 54A8: Structural properties of electrolyte solutions, lipid bilayers, and proteins. J. Chem. Theory Comput. 2013, 9, 1247–1264. 10.1021/ct300874c. 23418406 PMC3572754

[ref24] Sundaram V.; Lyulin A. V.; Baumeier B. Development and Testing of an All-Atom Force Field for Diketopyrrolopyrrole Polymers with Conjugated Substituents. J. Phys. Chem. B 2020, 124, 11030–11039. 10.1021/acs.jpcb.0c06787. 33211500 PMC7720275

[ref25] Malde A. K.; Zuo L.; Breeze M.; Stroet M.; Poger D.; Nair P. C.; Oostenbrink C.; Mark A. E. An Automated Force Field Topology Builder (ATB) and Repository: Version 1.0. J. Chem. Theory Comput. 2011, 7, 4026–4037. 10.1021/ct200196m. 26598349

[ref26] Singh U. C.; Kollman P. A. An approach to computing electrostatic charges for molecules. J. Comput. Chem. 1984, 5, 129–145. 10.1002/jcc.540050204.

[ref27] Moghe D.; Dutta G. K.; Patil S.; Guha S. Photocurrent spectroscopic studies of diketopyrrolopyrrole-based statistical copolymers. Phys. Chem. Chem. Phys. 2014, 16, 4291 10.1039/c3cp54644f. 24452360

[ref28] Liu F.; Gu Y.; Wang C.; Zhao W.; Chen D.; Briseno A. L.; Russell T. P. Efficient polymer solar cells based on a low bandgap semi-crystalline DPP polymer-PCBM blends. Adv. Mater. 2012, 24, 3947–3951. 10.1002/adma.201200902. 22689152

[ref29] Berendsen H. J.; Postma J. P.; Van Gunsteren W. F.; Dinola A.; Haak J. R. Molecular dynamics with coupling to an external bath. J. Chem. Phys. 1984, 81, 3684–3690. 10.1063/1.448118.

[ref30] Bussi G.; Donadio D.; Parrinello M. Canonical sampling through velocity rescaling. J. Chem. Phys. 2007, 126, 014101 10.1063/1.2408420. 17212484

[ref31] Darden T.; York D.; Pedersen L. Particle mesh Ewald: An *N* log(*N*) method for Ewald sums in large systems. J. Chem. Phys. 1993, 98, 10089–10092. 10.1063/1.464397.

[ref32] Brehm M.; Kirchner B. TRAVIS - A free analyzer and visualizer for monte carlo and molecular dynamics trajectories. J. Chem. Inf. Model. 2011, 51, 2007–2023. 10.1021/ci200217w. 21761915

[ref33] Brehm M.; Thomas M.; Gehrke S.; Kirchner B. TRAVIS—A free analyzer for trajectories from molecular simulation. J. Chem. Phys. 2020, 152, 164105 10.1063/5.0005078. 32357781

[ref34] Ngo T. T.; Nguyen D. N.; Nguyen V. T. Glass transition of PCBM, P3HT and their blends in quenched state. Adv. Nat. Sci.: Nanosci. Nanotechnol. 2012, 3, 045001 10.1088/2043-6262/3/4/045001.

[ref35] Guilbert A. A.; Zbiri M.; Dunbar A. D.; Nelson J. Quantitative Analysis of the Molecular Dynamics of P3HT:PCBM Bulk Heterojunction. J. Phys. Chem. B 2017, 121, 9073–9080. 10.1021/acs.jpcb.7b08312. 28834430

[ref36] van der Spoel D.; Berendsen H. J. Molecular dynamics simulations of leu-enkephalin in water and DMSO. Biophys. J. 1997, 72, 2032–2041. 10.1016/S0006-3495(97)78847-7. 9129806 PMC1184398

