# Supplementary material for: Effect of Solvent Removal Rate and Annealing on the Interface Properties in a Blend of a Diketopyrrolopyrrole-Based Polymer with Fullerene
Source: J Phys Chem B. 2022 Sep 19;126(38):7445–53. doi: 10.1021/acs.jpcb.2c04609 (PMC9527757; doi:10.1021/acs.jpcb.2c04609)
Supplement: Supplementary file 1 — jp2c04609_si_001.pdf [file jp2c04609_si_001.pdf]

Supporting Information for "Effect of solvent removal rate and annealing on the interface properties in a blend of a diketopyrrolopyrrole based polymer with fullerene"

Vivek Sundaram,[1,2,3] Alexey V. Lyulin,[2] and Björn Baumeier[1,3,*]

[1] *Department of Mathematics and Computer Science, Eindhoven University of Technology, P.O. Box 513, 5600 MB Eindhoven, The Netherlands*

[2] *Soft Matter and Biological Physics group, Department of Applied Physics, Eindhoven University of Technology, P.O. Box 513, 5600 MB Eindhoven, The Netherlands*

[3] *Institute for Complex Molecular Systems, Eindhoven University of Technology, P.O. Box 513, 5600 MB Eindhoven, The Netherlands*

CONTENTS

S1. Table to summarize simulation conditions for all the model systems S2

S2. Solvent orientation around the DPP units during evaporation S2

S3. Size effects on the local arrangement of polymer units and PCBM[60] S2

* b.baumeier@tue.nl

S1. TABLE TO SUMMARIZE SIMULATION CONDITIONS FOR ALL THE MODEL SYSTEMS

TABLE S1. Simulation conditions as used in the manuscript

	$I_a(120\,ns^{-1})$	$I_b(24\,ns^{-1})$	$I_c(6\,ns^{-1})$	II (Annealing)
# DPP2Py2T	6	6	6	6
# PCBM[60]	24	24	24	24
Initial # $CHCl_3$	12000	12000	12000	-
Initial temperature[K]	300	300	300	700
Final temperature[K]	300	300	300	300
Initial box size[nm]	12.0	12.0	12.0	4.0
Final box size[nm]	3.82	3.82	3.82	3.81
Denisty[kg/m^3]	1285	1286	1286	1305

S2. SOLVENT ORIENTATION AROUND THE DPP UNITS DURING EVAPORATION

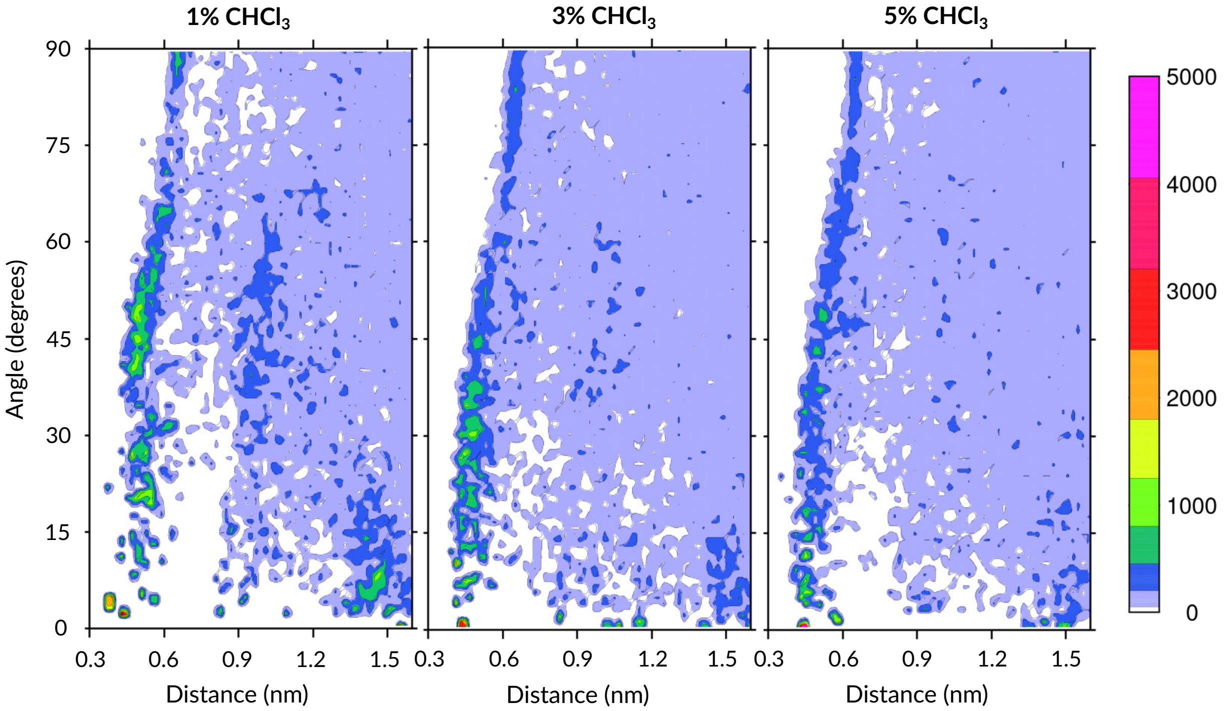

FIG. S1. Comparison of local arrangements of chloroform molecules with respect to the DPP unit of DPP2Py2T at 5%, 3% and 1% of the original quantity, from the simulation with $k_r^{\text{eff}} = 6\,\text{s}^{-1}$.

In order to evaluate the final orientation of the side-chain we study how the solvent is aligned around the DPP unit of the polymer along the final stages of the solvent evaporation. In particular we study when the solvent concentration in the mixture is 5%, 3% and 1% of the original quantity. In Figure S1 we see that the solvent has maximum concentration around the DPP units when the solvent concentration is 5%. As the simulation progresses the side-chains expand and occupy the space around the DPP unit thereby reducing the solvent concentration in closest vicinity to DPP which is evident from the scattered areas of solvent concentration as one goes from 5% to 1%.

S3. SIZE EFFECTS ON THE LOCAL ARRANGEMENT OF POLYMER UNITS AND PCBM[60]

To ascertain that loacl arrangement of the polymer units and PCBM[60] are not subject to system size effects, we have performed additional simulations of systems with twice the number of molecules (12 DPP2Py2T, 48 PCBM)

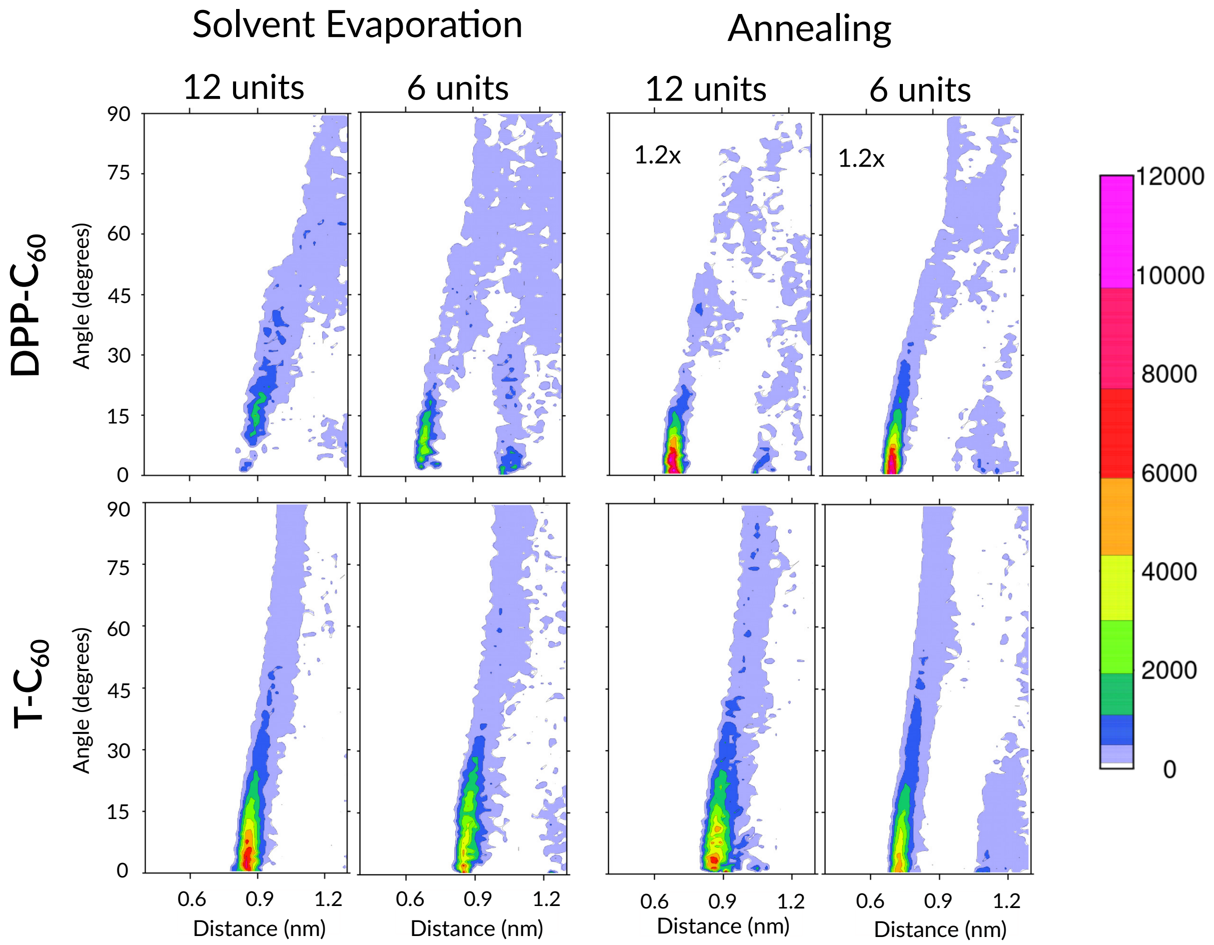

FIG. S2. Comparison of local arrangements for DPP-C60 and T-C60 for two different sizes of the system: (6 DPP2Py2T + 24 PCBM[60]) and (12 DPP2Py2T + 48 PCBM[60]).

as the one reported in the paper. Due to the computational cost of such simulations, we restrict ourselves to the comparison of an annealing run to a solvent evaporation run with an effective removal rate of (240 #/ns for the smaller system and 480 #/ns for the larger system), which is notably even faster than the fastest one reported in the manuscript.

Figure S2, shows the combined radial and angular distribution plots for the type as in Figure 7 of the manuscript (12 units: doubled system size, 6 units: system size as in the manuscript). While there are as expected some differences in the finer details of the arrangements, we emphasize that the observed different types of local arrangements between solvent evaporation and annealing protocols, respectively, are also found in the larger system.
